# Supplementary material for: An NlpC/P60 protein catalyzes a key step in peptidoglycan recycling at the intersection of energy recovery, cell division and immune evasion in the intracellular pathogen Chlamydia trachomatis
Source: PLoS Pathog. 2023 Feb 2;19(2):e1011047. doi: 10.1371/journal.ppat.1011047 (PMC9928106; doi:10.1371/journal.ppat.1011047)
Supplement: S1 Text — Supplementary Figures: Fig A. Primary sequence alignment of YkfCBce, YkfCCtr, YkfCCpn and NlpC/P60Ela. Fig B. Growth comparison of the E. coli ΔdapDΔmpl double mutant containing either the empty vector or C. trachomatis YkfC constructs. Fig C. Purification and in vitro activity of recombinant YkfCCtr. Fig D. In vitro activity of YkfCCtr on B. subtilis tripeptide. Fig E. In vitro activity of YkfCCtr on (non)PGN-derived peptides. Fig F. TLC analysis of the inhibitory effect of chloroacetone on YkfCCtr activity at molar inhibitor:protein ratios below 8:1. Fig G. Viability of HEp-2 cells upon treatment with cysteine protease inhibitors. Fig H. Wild-type YkfCCtr expression levels are required for optimal chlamydial growth. Fig I. Chlamydial inclusion formation is not affected by overexpression of YkfCCtr_6xH. Fig J. Effect of the cysteine protease inhibitor chloroacetone on uninfected HEK-Blue hNOD1 cells. Fig K. Growth comparison of the E. coli ΔdapDΔmpl double mutant containing either the empty vector or NlpC/P60 constructs. Fig L. 3D in silico models of chlamydial NlpC/P60 enzymes. Fig M. Primary sequence alignment of YkfCBce and NlpC/P60Sne. Fig N. (a) Quantification of bacterial growth by 16S qPCR for E. lausannensis and S. negevensi. Antibodies raised against NlpC/P60 of E. lausannensis (b) and S. negevensis (c) specifically label bacteria. Supplementary Material and Methods: Cloning of C. trachomatis, Immunofluorescence and confocal microscopy of C. trachomatis, RT-qPCR analysis, IFU assay, Cytotoxicity assay. Supplementary Tables: Table A in S1 Text. Strains, plasmids and oligonucleotides used in this study. (PDF) [file ppat.1011047.s002.pdf]

Supplementary Information for:

An NlpC/P60 protein catalyzes a key step in peptidoglycan recycling at the intersection of energy recovery, cell division and immune evasion in the intracellular pathogen *Chlamydia trachomatis*

Jula Reuter[¶], Christian Otten[¶*], Nicolas Jacquier, Junghoon Lee, Dominique Mengin-Lecreulx, Iris Löckener, Robert Kluj, Christoph Mayer, Federico Corona, Julia Dannenberg, Sébastien Aeby, Henrike Bühl, Gilbert Greub, Waldemar Vollmer, Scot P. Ouellette, Tanja Schneider, Beate Henrichfreise[*]

¶ These authors contributed equally to this work.
[*] Corresponding authors

Supplementary Figures

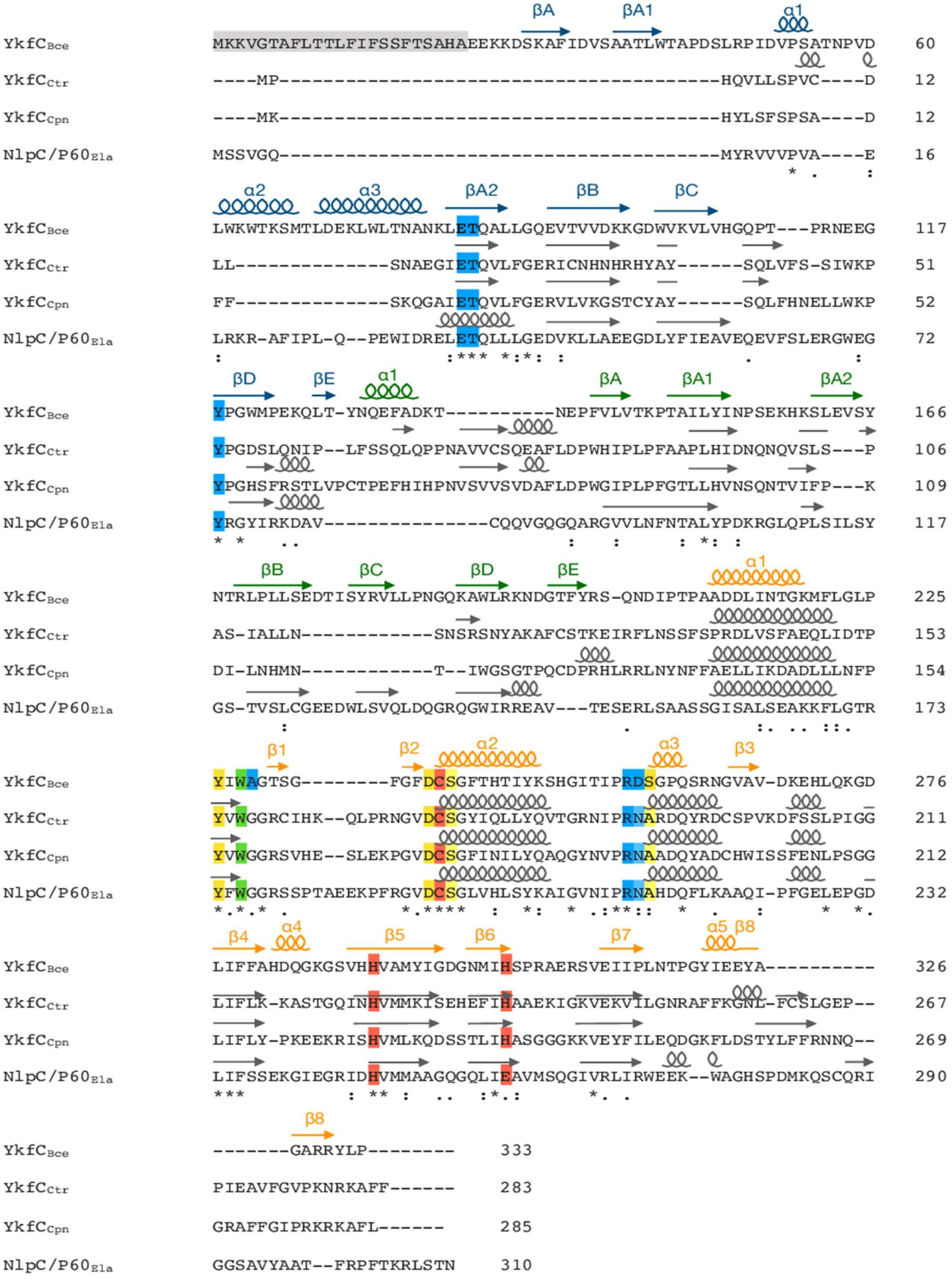

Fig A in S1 Text. Primary sequence alignment of YkfC$_{Bce}$, YkfC$_{Ctr}$, YkfC$_{Cpn}$ and NlpC/P60$_{Ela}$. The secondary structure of YkfC$_{Bce}$ characterized by Xu *et al.,* 2010 [1] is indicated above its sequence with the Sh3b1 domain in blue, the Sh3b2 domain in green and the NlpC/P60 domain in orange. The *in silico* predicted secondary structures of the chlamydial proteins are shown in grey above the respective sequences. The catalytic triad comprising Cys, His and a polar residue is shown in red, residues contributing to the S1 and S2 sites essential for substrate recognition in YkfC$_{Bce}$ are highlighted in yellow and blue, respectively. Residues contributing to both sites are further highlighted in green. The signal peptide sequence of YkfC$_{Bce}$ is displayed in grey.

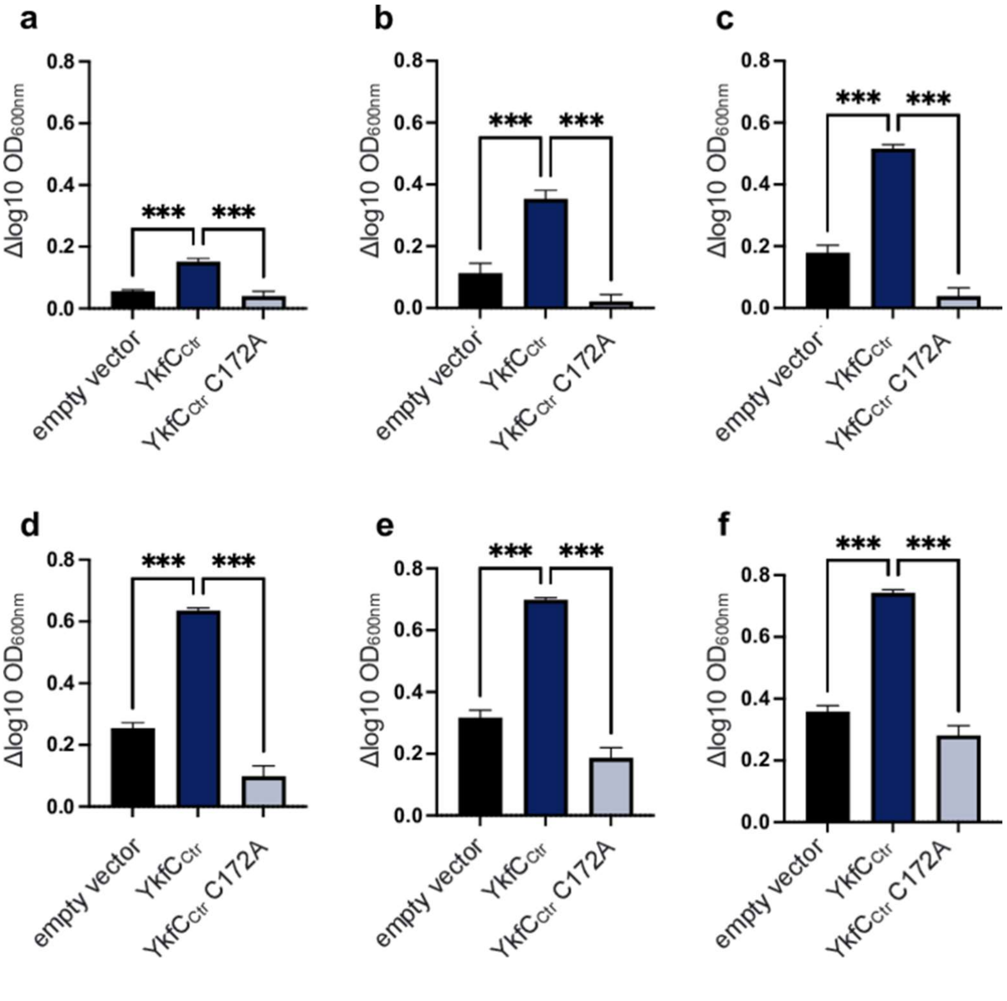

Fig B in S1 Text. Growth comparison of *E. coli* Δ*dapD* Δ*mpl* double mutant containing either the empty vector or *C. trachomatis* YkfC constructs. Growth at (a) 1 h, (b) 2 h, (c) 3 h, (d) 4 h, (e) 5 h and (f) 6 h was compared by one-way ANOVA analysis. P-values are shown as asterisks: n.s.: $P \geqslant 0.05$, *: P = 0.01 to 0.05, **: P = 0.001 to 0.01, ***: $P < 0.001$. Error bars indicate ± s.d. (n=3).

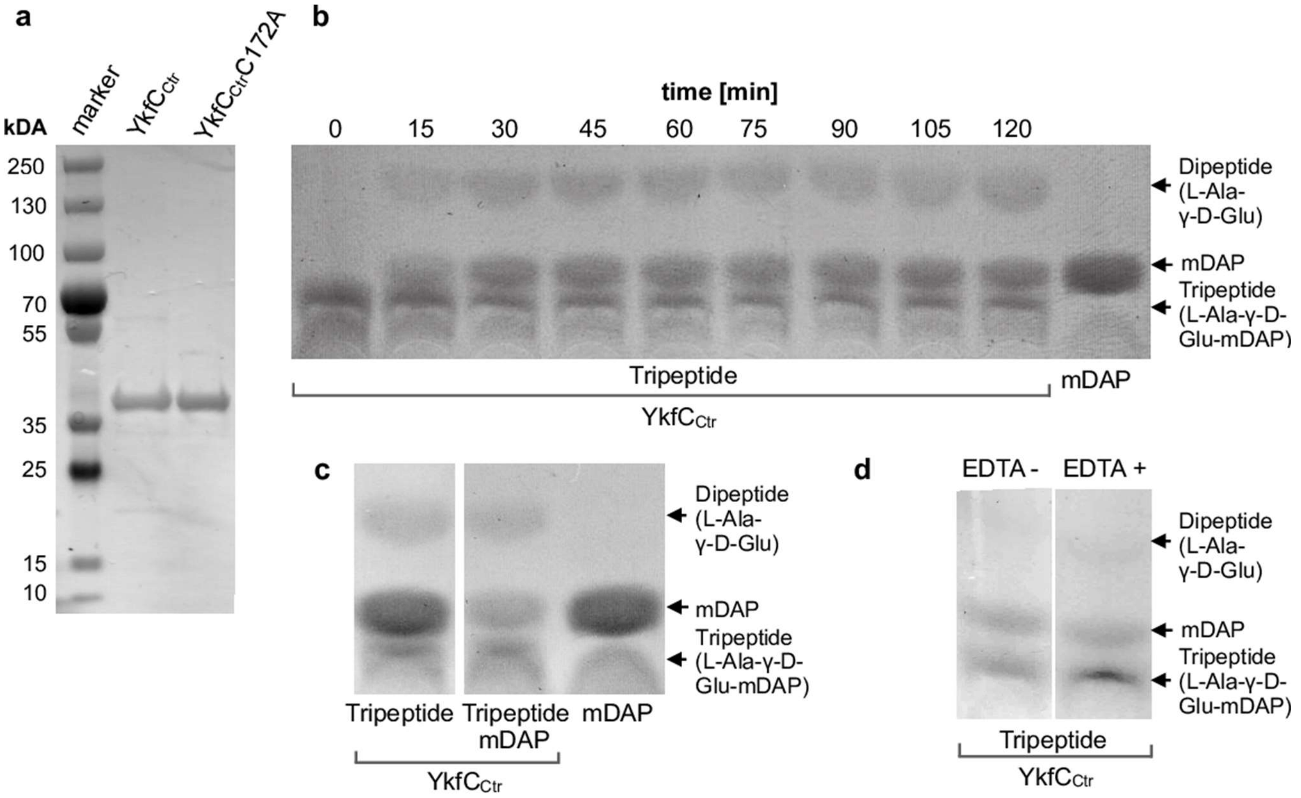

Fig C in S1 Text. Purification and *in vitro* activity of recombinant YkfC$_{\mathrm{Ctr}}$. Recombinant YkfC$_{\mathrm{Ctr}}$ as well as its active site mutant YkfC$_{\mathrm{Ctr}}$C172A were analyzed by SDS-PAGE **(a)**. *In vitro* peptidase activity over time was analyzed by TLC using L-Ala-γ-D-Glu-mDAP tripeptide as substrate **(b)**. YkfC$_{\mathrm{Ctr}}$ activity was time dependent reaching a plateau at t=30 min and was not decreased in presence of the reaction product mDAP (c) or the chelator EDTA **(d)**.

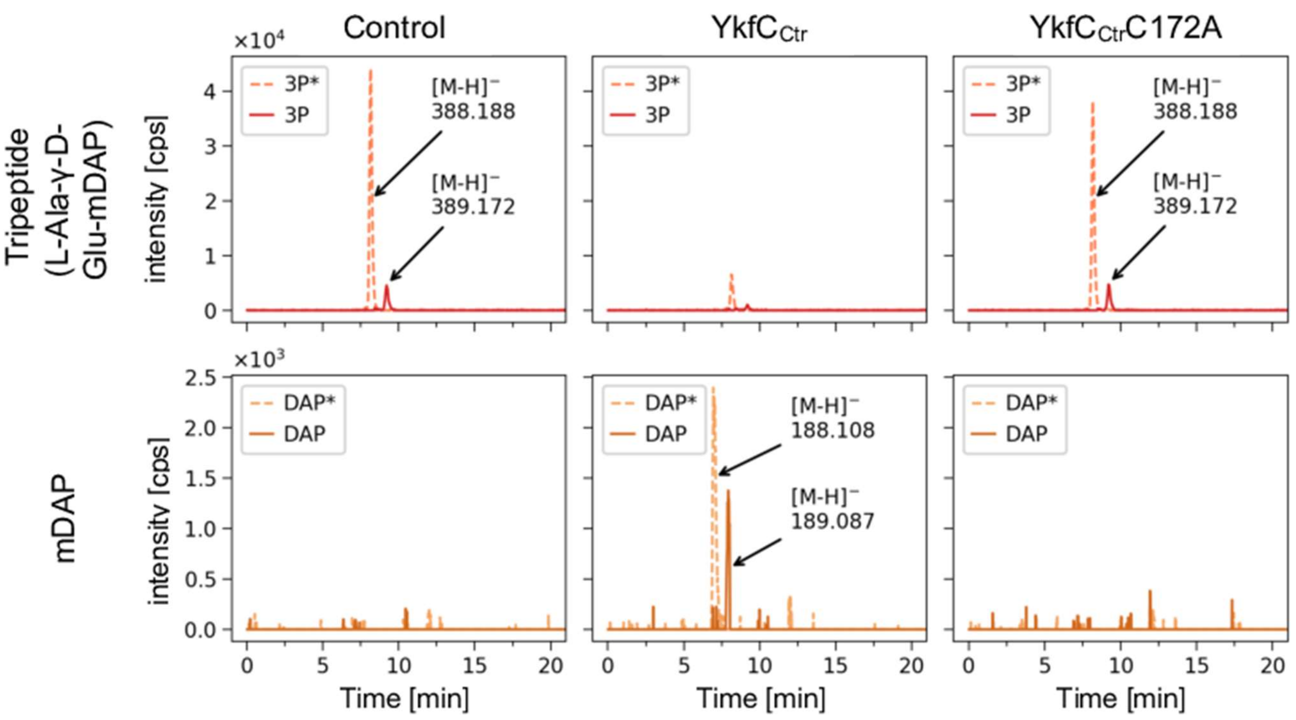

Fig D in S1 Text. *In vitro* activity of YkfC$_{Ctr}$ on *B. subtilis* tripeptide. LC-MS analysis of YkfC$_{Ctr}$ activity on CwlC derived amidated tripeptide from *B. subtilis* PGN. YkfC$_{Ctr}$, but not its active-site mutant YkfC$_{Ctr}$C172A, was active on this substrate regardless of the amidation of the free carboxyl group in mDAP as shown by the release of amidated mDAP. Data presented as extracted ion chromatograms for theoretical masses of amidated tripeptide (3P*, $[M-H]^- = 388.183$), tripeptide (3P, $[M-H]^- = 389.167$), amidated DAP (DAP*, $[M-H]^- = 188.103$) and DAP ($[M-H]^- = 189.087$) within an error of ± 0.02.

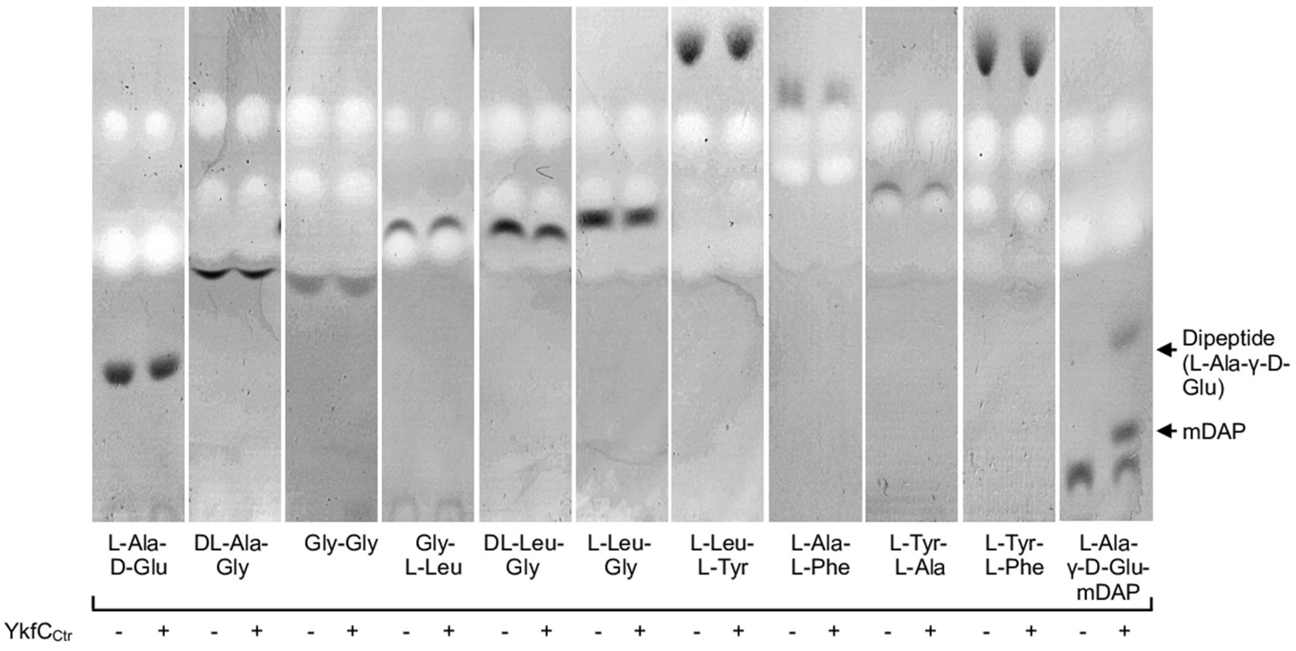

Fig E in S1 Text. *In vitro* activity of YkfC$_{Ctr}$ on (non)-PGN derived peptides. *In vitro* peptidase activity towards 10 (non)-PGN-derived peptides was analyzed by TLC. None of the peptides was used as asubstrate by YkfCCtr. L-Ala-γ-D-Glu-mDAP was used as positive control.

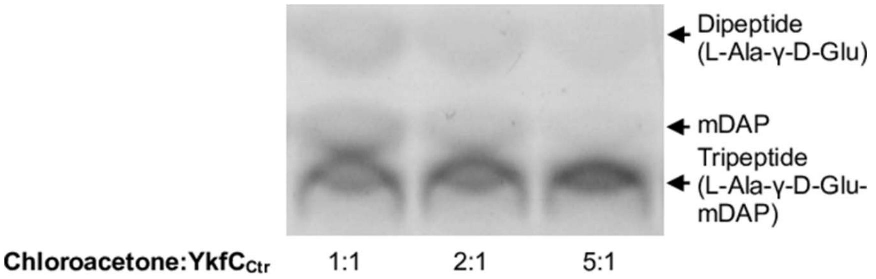

Fig F in S1 Text. TLC analysis of the inhibitory effect of chloroacetone on YkfC$_{Ctr}$ activity at molar inhibitor:protein ratios below 8:1 (corresponding to a molar ratio inhibitor:active site cysteine of 1:1, provided that the additional seven cysteines in YkfC$_{Ctr}$ are accessible for the alkylating chemical). YkfC$_{Ctr}$ retained activity at all tested inhibitor:protein ratios below 8:1 as shown by the presence of reaction product mDAP.

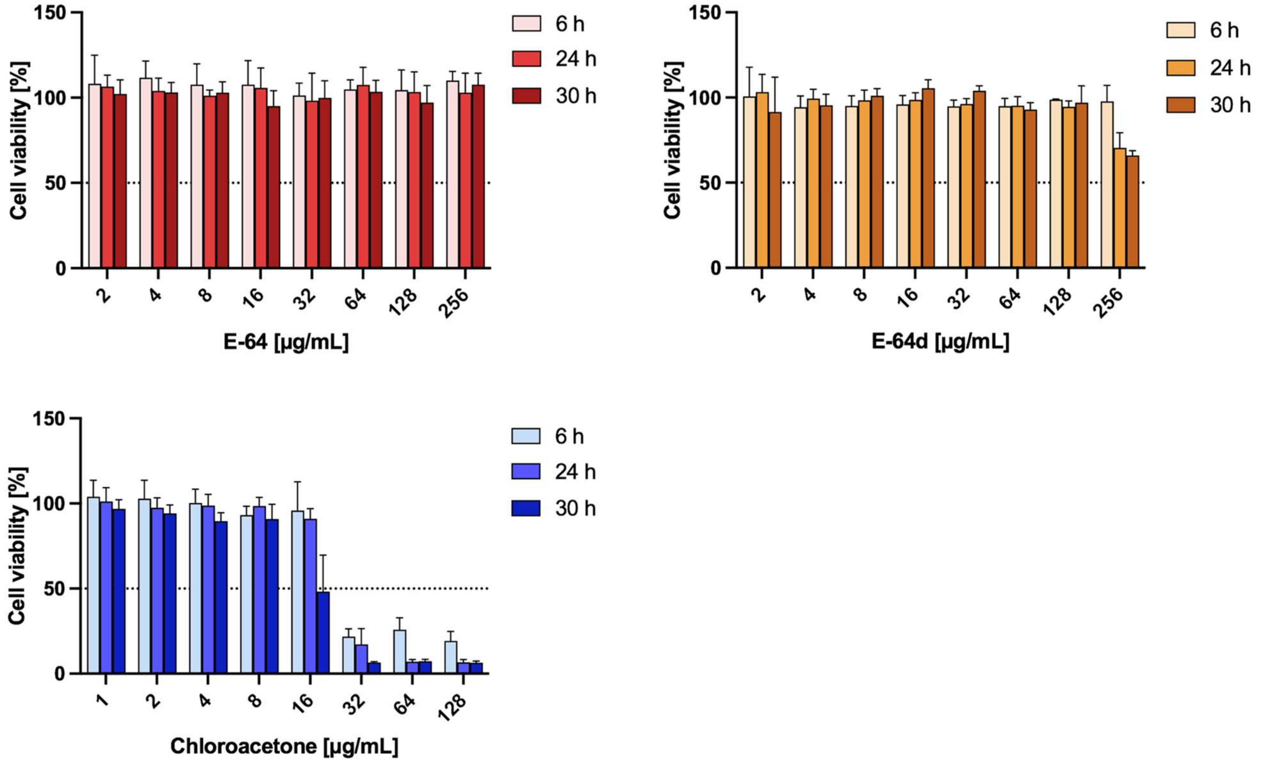

Fig G in S1 Text. Viability of HEp-2 cells upon treatment with cysteine protease inhibitors. Inhibitors were tested on confluent HEp-2 monolayer for 6 h, 24 h or 30 h. Cytotoxicity was visualized using alamarBlue cell viability reagent. Error bars indicate ± s.d.

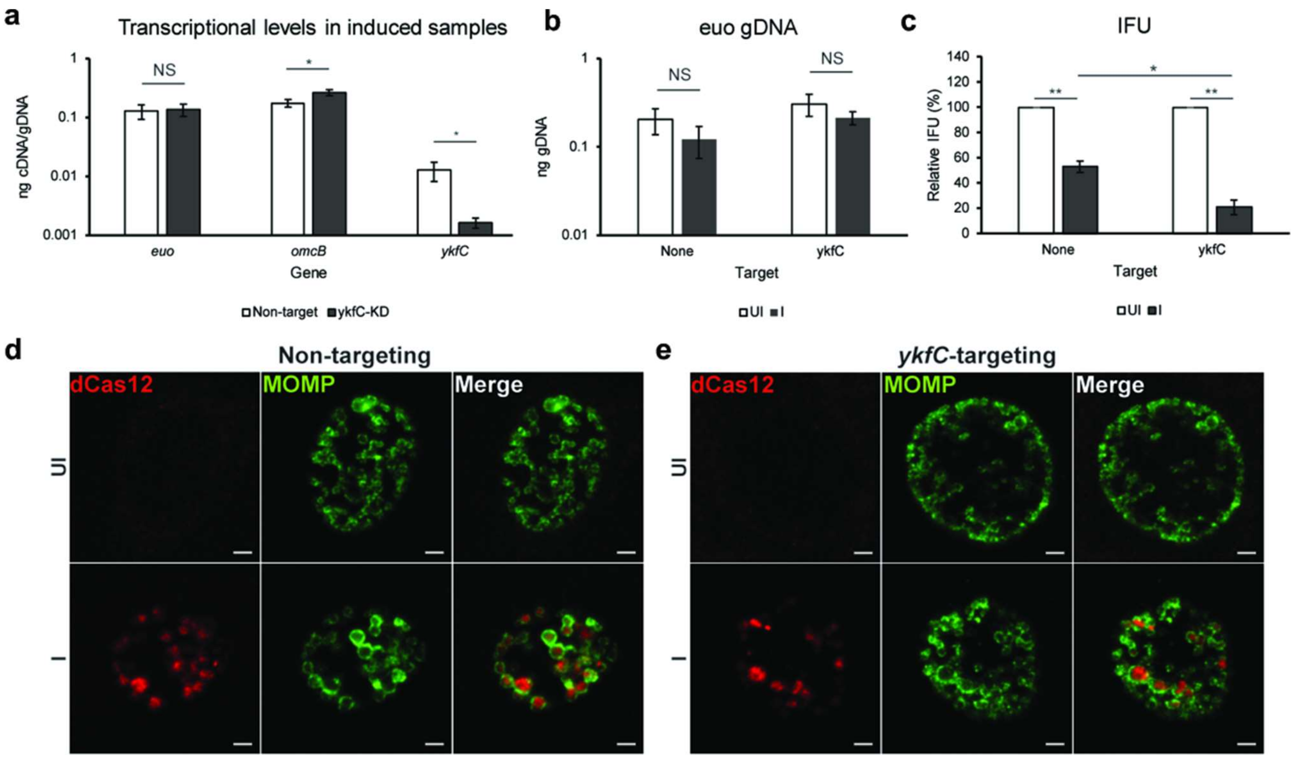

Fig H in S1 Text. Wild-type YkfC$_{Ctr}$ expression levels are required for optimal chlamydial growth. Constructs encoding dCas12-crRNA CRISPRi system targeting *ykfC$_{Ctr}$* or a non-targeting control were transformed into *C. trachomatis* (-pL2). McCoy cells were infected with these transformants, and dCas12 expression was induced or not with 10 nM aTc at 4 hpi. At 24 hpi, RNA and genomic DNA were isolated and used for (RT)-qPCR to measure transcript and genomic DNA levels. **(a)** Transcript levels of *euo*, *omcB*, and *ykfC* in dCas12-induced conditions at 24 hpi in the non-targeting or *ykfC$_{Ctr}$*-targeting knockdown strains. **(b)** The levels of genomic DNA of non-targeting and *ykfC$_{Ctr}$*-targeting knockdown strains at 24 hpi in uninduced (UI) or induced (I) conditions. **(c)** Inclusion forming units of non-targeting and *ykfC$_{Ctr}$*-targeting knockdown strains at 24 hpi in uninduced and induced conditions. **(a-c)** Error bars represent ± standard deviation (n = 3). *: p<0.05; **: p<0.001 **(d, e)** IFA controls of non-targeting and *ykfC$_{Ctr}$*-targeting knockdown strains at 24 hpi. MOMP (green) and dCas12 (red) were stained. The images were acquired on a Zeiss AxioImager.Z2 equipped with an Apotome2 using a 100X objective. Scale bar: 2 µm.

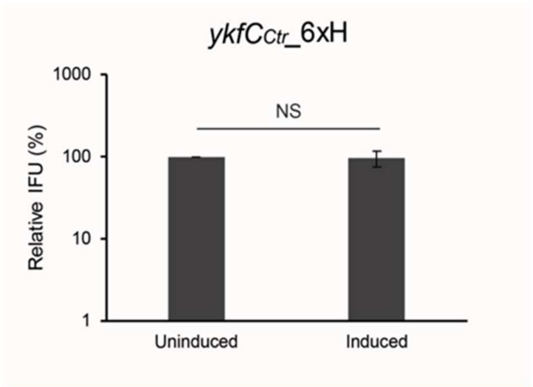

Fig I in S1 Text. Chlamydial inclusion formation is not affected by overexpression of YkfC$_{Ctr}$_6xH. The effect of YkfC$_{Ctr}$_6xH overexpression on inclusion formation was tested by inclusion forming unit (IFU) assay. McCoy cells were infected with uninduced and induced IFU samples. At 24 hpi, the infected cells were fixed, stained with goat anti-MOMP, and the inclusion number was counted. NS: not significant.

a

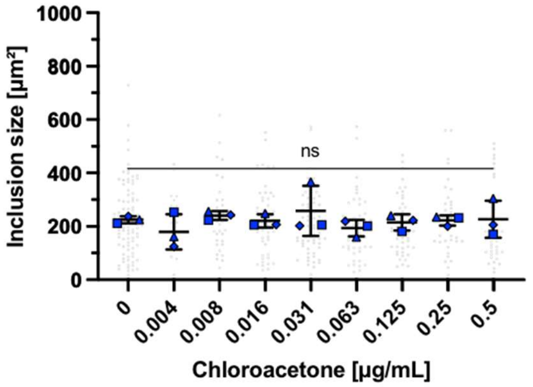

b

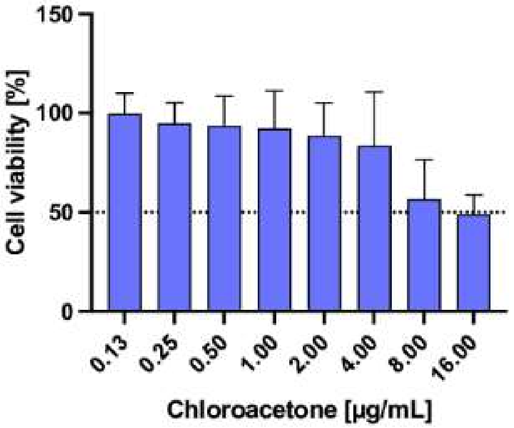

c

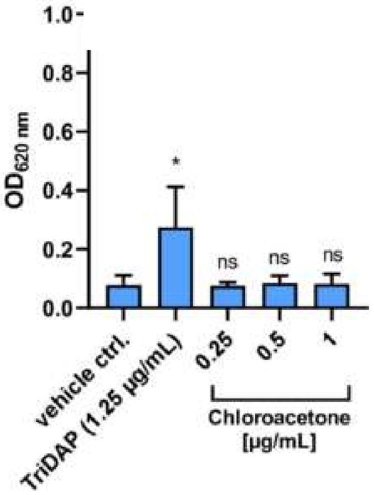

Fig J in S1 Text. Effect of cysteine protease inhibitor chloroacetone on the inclusion size of *C. trachomatis* exposed to concentrations below the MIC (4 µg/mL) and on uninfected HEK-Blue hNOD1 cells. (a) Effect on inclusion size. Chloroacetone did not impact on inclusion size in the tested sub-MIC range of concentrations (0.004-0,5 µg/mL) including concentrations that exerted an increase in NOD1 activation (0.016-0,031 µg/mL). **(b)** Effect on cell viability**.** Chloroacetone was tested in a concentration range of 0.13-16 µg/mL for 24 h treatment duration on uninfected HEK-Blue hNOD1 cells. Cytotoxicity was analyzed using alamarBlue cell viability reagent. Error bars indicate ± s.d. (n=4). **(c)** Effect on NOD1 stimulation. Chloroacetone was tested on uninfected HEK-Blue hNOD1 cells in a concentration range of 0.25-1 µg/mL for 18h. Stimulation of NOD1 was measured at $OD_{620\ nm}$. TriDAP was used as positive control. Error bars indicate ± s.d. (n=3).

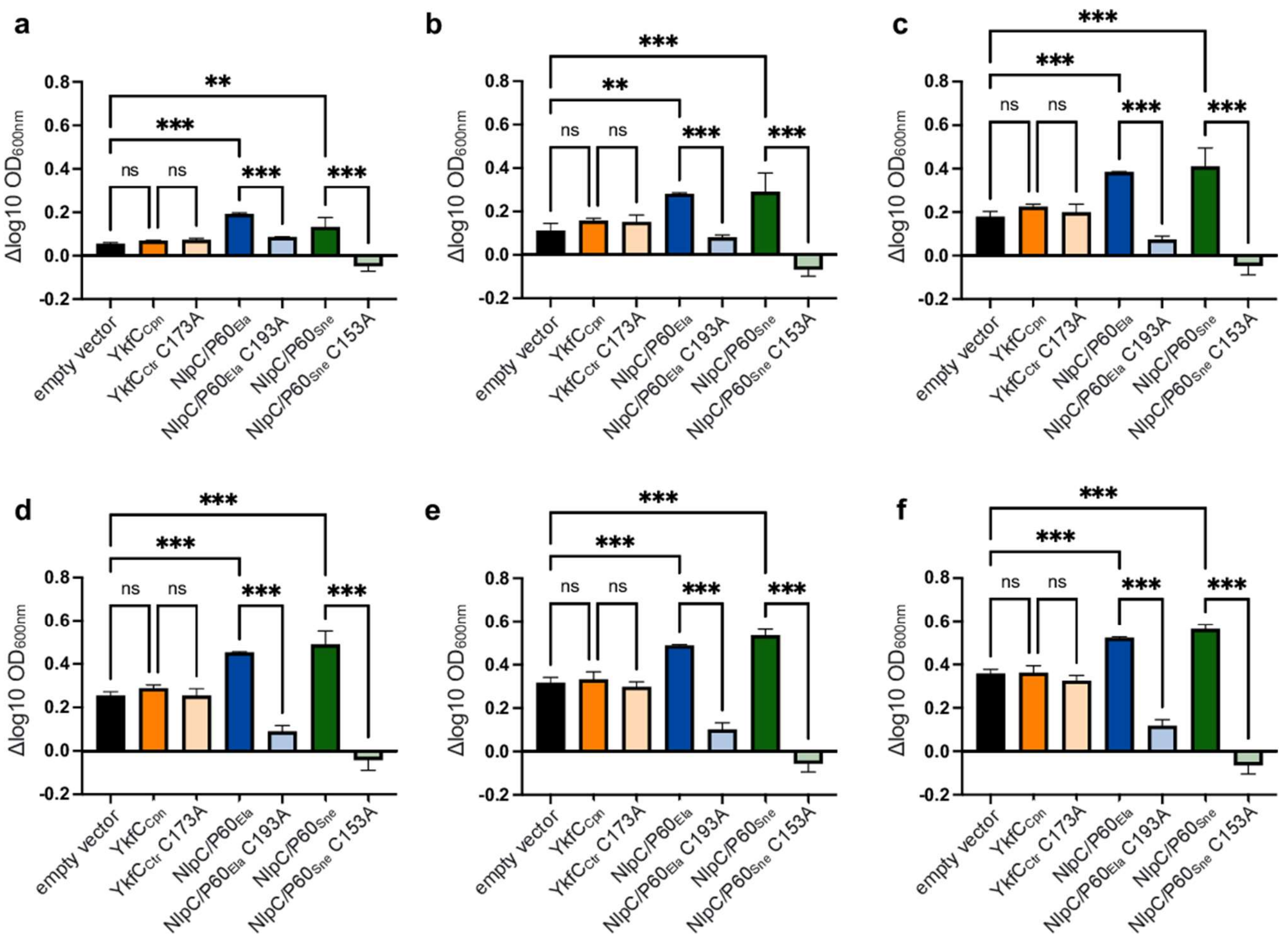

Fig K in S1 Text. Growth comparison of *E. coli* Δ*dapD* Δ*mpl* double mutant containing either the empty vector or chlamydial NlpC/P60 constructs. Growth at (a) 1 h, (b) 2 h, (c) 3 h, (d) 4 h, (e) 5 h and (f) 6 h was compared by one-way ANOVA analysis. P-values are shown as asterisks: n.s.: P ⩾ 0.05, *: P = 0.01 to 0.05, **: P = 0.001 to 0.01, ***: P < 0.001. Error bars indicate ± s.d. (n=3-6).

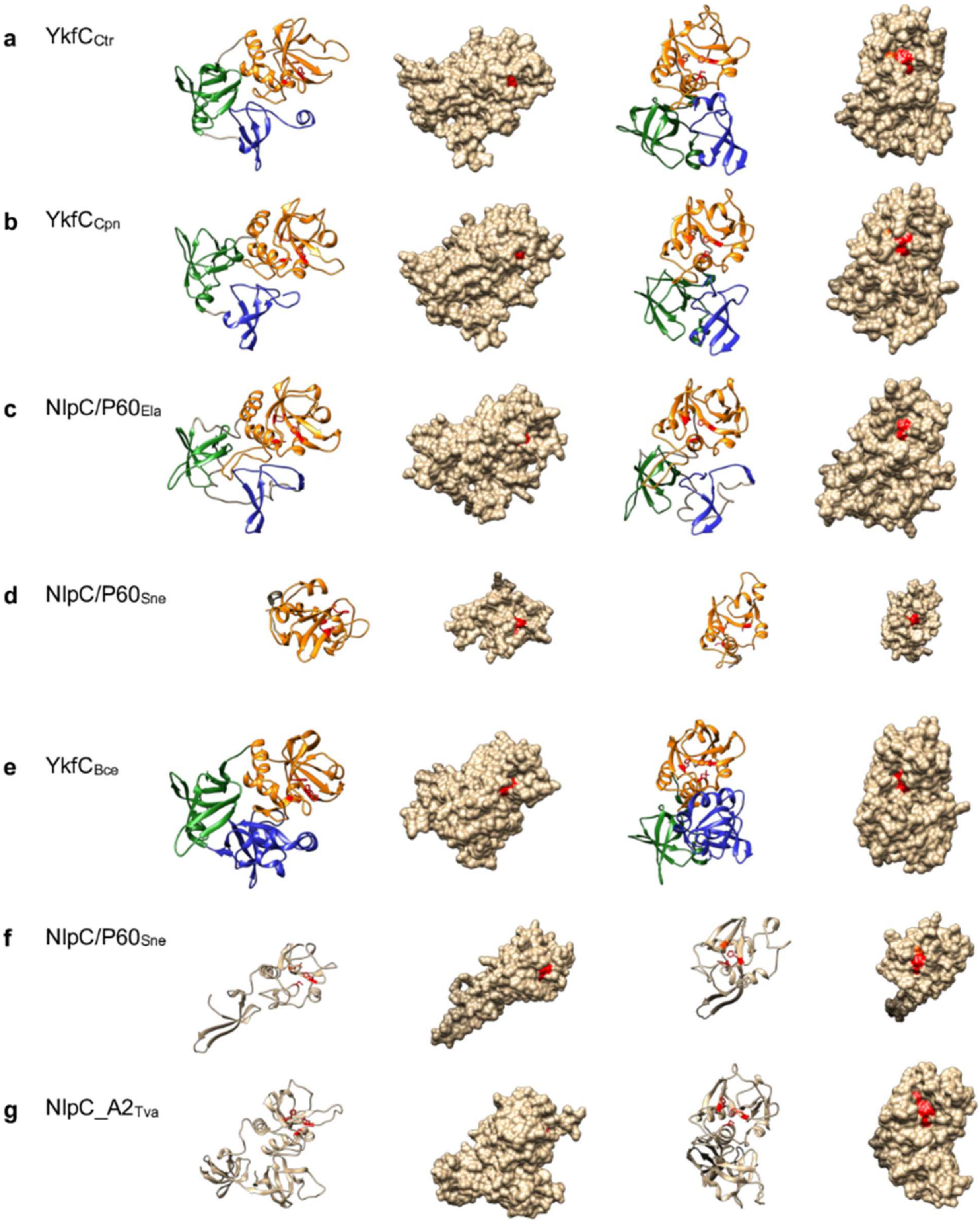

Fig L in S1 Text. 3D *in silico* models of chlamydial NlpC/P60 enzymes. The 3D structures of YkfC_{Ctr} (**a**), YkfC_{Cpn} (**b**), $\text{NlpC/P60}_{\text{Ela}}$ (**c**) and $\text{NlpC/P60}_{\text{Sne}}$ (**d**) were modeled based on Phyre2 alignment against the structure of YkfC_{Bce} (**e**) characterized by Xu *et al*., 2010 [1]. The Sh3b1 domain is highlighted in blue, the Sh3b2 domain in green and the NlpC/P60 domain in orange. The catalytic triad comprising Cys, His and a polar residue is shown in red. For $\text{NlpC/P60}_{\text{Sne}}$ another 3 D structure was predicted based on Phyre2 alignment against the structure of *Trichomonas vaginalis* NlpC/P60 protein NlpCA2 ($\text{NlpC\_A2}_{\text{Tva}}$) characterized by Pinheiro *et al*., 2019 [2] because this protein showed highest sequence and secondary structure matches in the database with *Simkania* NlpC/P60 (**f,g**).

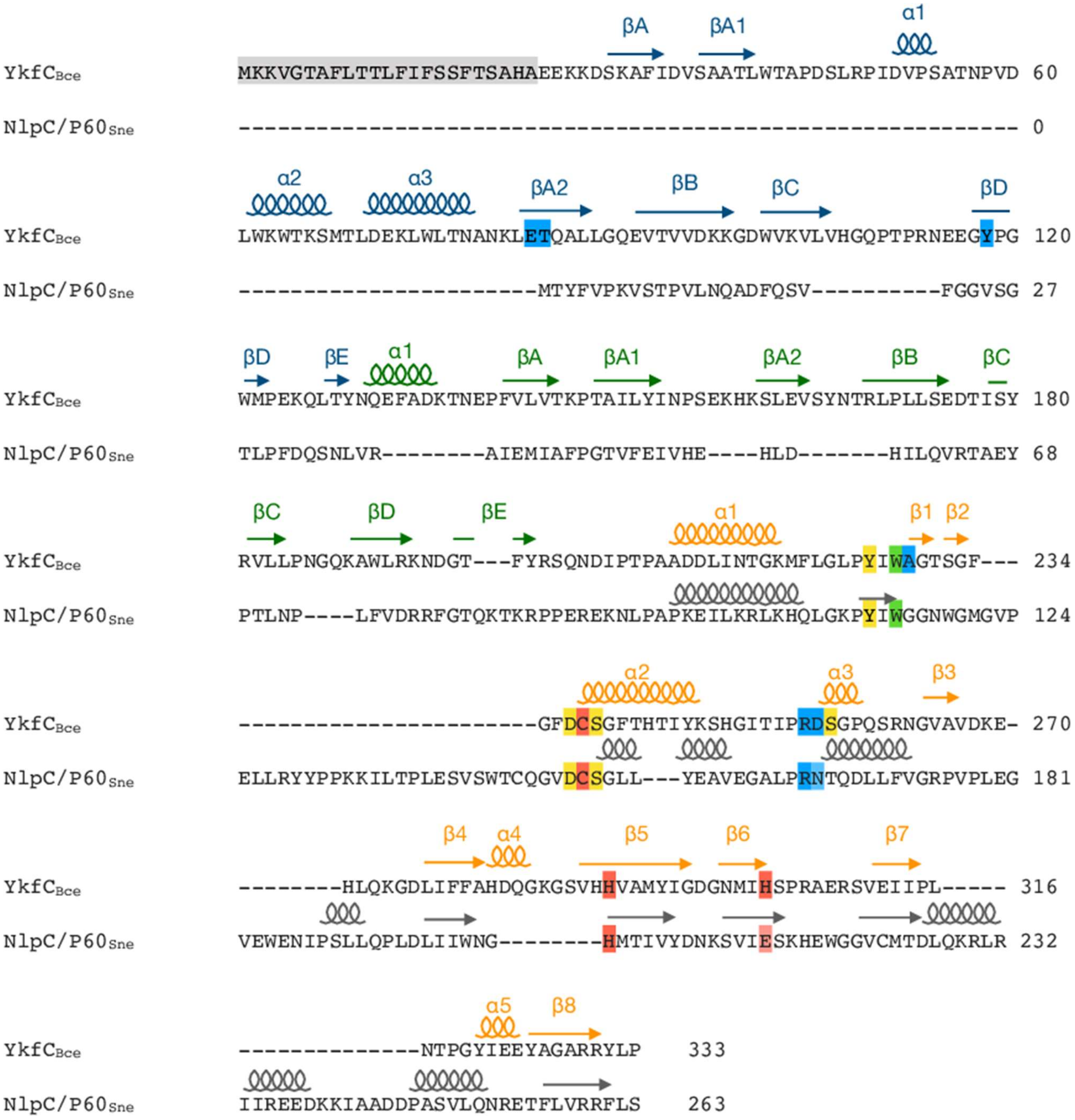

Fig M in S1 Text. Primary sequence alignment of YkfC$_{Bce}$ and NlpC/P60$_{Sne}$. The secondary structure of YkfC$_{Bce}$ characterized by Xu *et al.*, 2010 [1] is indicated above its sequence with the Sh3b1 domain in blue, the Sh3b2 domain in green and the NlpC/P60 domain in orange. The in silico predicted secondary structure of NlpC/P60$_{Sne}$ is shown in grey above the respective sequences. The catalytic triad comprising Cys, His and a polar residue is shown in red, residues contributing to the S1 and S2 sites essential for substrate recognition in YkfC$_{Bce}$ are highlighted in yellow and blue, respectively. Residues contributing to both sites are further highlighted in green. The signal peptide sequence of YkfC$_{Bce}$ is displayed in grey.

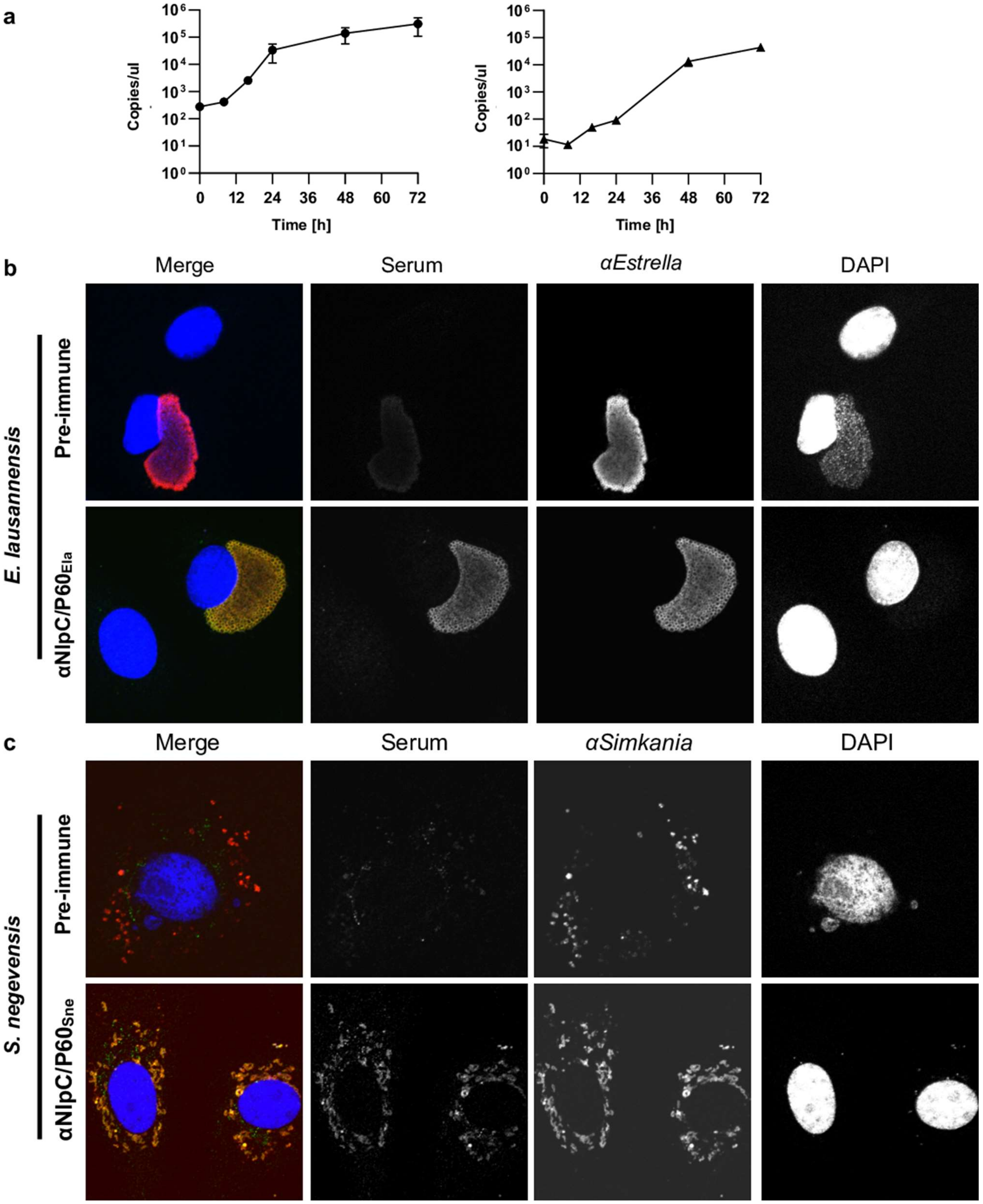

Fig N in S1 Text. (a) Quantification of bacterial growth by 16S qPCR for *E. lausannensis* and *S. negevensis*. DNA was extracted and copy number of bacterial genomes was quantified by qPCR targeting the 16S gene. This quantification was used to normalize the RNA quantification according to the number of bacteria in the sample. Error bars indicate ± s.d. (n=4). **Antibodies raised against NlpC/P60 of *E. lausannensis* (b) and *S. negevensis* (c) specifically label bacteria.** Cells infected with *S. negevensis* or *E. lausannensis* were labelled with mice serum taken before (pre-immune) or after immunization with the indicated protein (green), with species-specific antibodies (red) and DAPI (blue). Bar = 2 µm.

Supplementary Material and Methods

Cloning of *C. trachomatis*. For *ctl0328* (homolog of *ct127*)-knockdown in *C. trachomatis* serovar L2 (strain 434/Bu), a gBlock (IDTDNA, Coralville, IA) encoding a crRNA cassette was designed to target the 5‘ intergenic region and inserted into pBOMBL12CRia linearized by BamHI using HiFi (NEB) as described [3]. As a control, a scrambled non-targeting crRNA gBlock cassette was also inserted into the BamHI-digested pBOMBL12CRia empty vector. The HiFi products were transformed into NEB10β competent cells (NEB) and plated on LB agar containing 100 µg/mL ampicillin.

Immunofluorescence and confocal microscopy of *C. trachomatis*. For IFA controls of qPCR, McCoy cells were infected with the transformants, and the infected cells were fixed with 100% MeOH for 10 min. The coverslips were stained with primary antibody goat anti-major outer membrane protein (MOMP) of *C. trachomatis* (Meridian, Memphis, TN) and mouse anti-dCas12 (Millipore-Sigma). Subsequently, the coverslips were stained with secondary antibodies donkey anti-goat (488) or donkey anti-mouse (594) (Invitrogen).

RT-qPCR analysis. McCoy cells were infected with the *C. trachomatis* L2 transformants carrying the aTc-inducible constructs encoding dCas12 with or without constitutively expressed crRNA targeting $ykfC_{Ctr}$ or the non-targeting control. At 4hpi, the constructs were induced or not with 10 nM aTc. RNA and genomic DNA were collected using Trizol (Invitrogen) and DNeasy Tissue (Qiagen) kit as described previously [4]. The isolated RNA was treated with DNase to remove DNA contamination, and cDNA was synthesized with the DNased RNA using Superscript III reverse transcriptase (Invitrogen). Equal volumes of cDNA were used in qPCR reactions [4]. Similarly, equal mass of DNA was used from each sample in qPCR reactions using the primers listed in

Supplementary Table 1 to quantify chlamydial genomes, which were used to normalize the amount of cDNA.

IFU assay. The transformants were infected into McCoy cell monolayers in 6-well plate (MOI=0.1). At 4 hpi, the contructs were induced with 0 or 10 nM aTc, and the transformants were harvest at 24 hpi and frozen at -80°C. The IFU samples were thawed and diluted to 10^{-4}. The diluted cells were infected into McCoy cells in 24-well plates. At 24 hpi, the infected cells were fixed and stained with goat anti-MOMP (594), and the number of inclusions was counted.

Cytotoxicity assay. Cytotoxicity of cysteine protease inhibitors on HEp-2 and HEK-Blue hNOD1 cells, was assessed via the resazurin-based alamarBlue cell viability reagent (Thermo Fisher Scientific, Waltham, MA). $5x10^4$ HEp-2 cells/mL or $2x10^5$ HEK-Blue hNOD1 cells/mL were seeded into flat TC-96-well plates (Sarstedt, Nümbrecht, Germany) and incubated 2 days for HEp-2 and 20 h for HEK-Blue hNOD1 cells at 37 °C and 5% CO_2. Next, cells were washed with medium followed by the addition of inhibitors in a serial dilution within the indicated concentration range. After 6, 24 or 30 h incubation with the compound, cells were washed twice with Hanks' balanced salt solution (HBSS) and incubated with alamarBlue cell viability reagent diluted 1:10 in HBSS for 1 h. Convertion of resazurin into resorufin by viable cells was determined by transferring the supernatant into black 96-well plates (Greiner Bio-One, Frickenhausen, Germany) and measuring the fluorescence at 550 nm excitation and 595 nm emission wavelength using a Tecan infinite M200 plate reader and Tecan SparkControl software (Tecan Group, Männedorf, Switzerland).

Supplementary Tables

Table A in S1 Text. Strains, plasmids and oligonucleotides[a] used in this study.

Strain, plasmid or oligonucleotide	Description	Reference or source
E. coli BL21(DE3)	Expression strain	New England Biolabs
E. coli JW2163-3	*ΔmepS* mutant, used for complementation assays	Keio collection; Baba et al., 2006 [5]
E. coli Δ*dapD* Δ*mpl*	*ΔmplΔdapD* double mutant, used for complementation assays	this study
C. trachomatis D/UW-3/CX (ATCC VR-885)	*C. trachomatis* strain used for MIC and hNOD1 activation experiments	ATCC
HEp-2 cells (ATCC CCL-23)	Used for propagation of *C. trachomatis* in MIC experiments	ATCC
HEK-Blue hNOD1 cells	Used for hNOD1 assay with *C. trachomatis*	InvivoGen, Toulouse, France
Vero cells (ATCC CCL-81)	Used for propagation of *Chlamydia*-like organisms	ATCC
pET52b-ykfC$_{Ctr}$	*ykfC* from *C. trachomatis*, N-terminal Strep-tag, used for cytoplasmic overproduction	this study
pET52b-ykfC$_{Ctr}$C172A	*ykfC* from *C. trachomatis*, N-terminal Strep-tag, YkfC$_{Ctr}$ C172A mutant (YkfC$_{Ctr}$C172A), used for cytoplasmic overproduction	this study
pBAD24+-ykfC$_{Ctr}$	*ykfC* from *C. trachomatis*, used for complementation assays	this study
pBAD24+ykfC$_{Ctr}$C172A	*ykfC* from *C. trachomatis*, YkfC$_{Ctr}$ C172A mutant (YkfC$_{Ctr}$C172A), used for complementation assays	this study
pBAD24+-ykfC$_{Cpn}$	*ykfC* from *C. pneumoniae*, used for complementation assays	this study
pBAD24+ykfC$_{Cpn}$C173A	*ykfC* from *C. pneumoniae*, YkfC$_{Cpn}$ C173A mutant (YkfC$_{Cpn}$C173A), used for complementation assays	this study
pBAD24+-nlpC/P60$_{Ela}$	*nlpC/P60* from *E. lausannensis*, used for complementation assays	this study
pBAD24+-nlpC/P60$_{Ela}$C193A	*nlpC/P60* from *E. lausannensis*, NlpC/P60$_{Ela}$ C193A mutant (YkfC$_{Ela}$C172A), used for complementation assays	this study
pBAD24+-nlpC/P60$_{Sne}$	*nlpC/P60* from *S. negevensis*, used for complementation assays	this study
pBAD24+-nlpC/P60$_{Sne}$C153A	*nlpC/P60* from *S. negevensis*, NlpC/P60$_{Sne}$ C153A mutant (YkfC$_{Sne}$C153A), used for complementation assays	this study
pBAD24+-mepS$_{Eco}$	*mepS* from *E. coli*, used for complementation assays	this study
ykfC$_{Ctr}$-pET52_fow	gcgcgcCCCGGGccgcaccaagtcttatt	this study
ykfC$_{Ctr}$-pET52_rev	gcgcgcCGGCCGtcaaaagaaggcttttctatttttag	this study

ykfC$_{Ctr}$-pBAD24+_fow	GAGGAATTCACCATGCCGCACCAAGTCTTATTGTCTCCT	this study
ykfC$_{Ctr}$-pBAD24+_rev	TCATCCGCCAAAACATCAAAAGAAGGCTTTTCTATTTTT	this study
ykfC$_{Cpn}$-pBAD24+_fow	GAGGAATTCACCATGAAACACTACCTATCATTTTCTCCT	this study
ykfC$_{Cpn}$-pBAD24+_rev	TCATCCGCCAAAACATTACAGAAAGGCTTTTCTTTTTCT	this study
nlpC/P60$_{Ela}$-pBAD24+_fow	GAGGAATTCACCATGAGTTCTGTCGGGCAGATGT	this study
nlpC/P60$_{Ela}$-pBAD24+_rev	TCATCCGCCAAAACATCAGTTGGTGGACAGTCTCTT	this study
nlpC/P60$_{Sne}$-pBAD24+_fow	GAGGAATTCACCATGACCTATTTTGTCCCAAAAGTTTCAA	this study
nlpC/P60$_{Sne}$-pBAD24+_rev	TCATCCGCCAAAACATTACGAGAGGAATCGTCGGACT	this study
mepS$_{Eco}$-pBAD24+_fow	GAG GAA TTC ACC ATG GTC AAA TCT CAA CCG ATT TTG AG	this study
mepS$_{Eco}$-pBAD24+_rev	TCA TCC GCC AAA ACA TTA GCT GCG GCT GAG AAC CC	this study
ykfC$_{Ctr}$-C172A_fow	acagcttcctcgtaatggtgtagatgcttcggggtatattc	this study
ykfC$_{Ctr}$-C172A_rev	gaatataccccgaagcatctacaccattacgaggaagctgt	this study
ykfC$_{Cpn}$-C173A_fow	agtctggaaaagccgggtgttgatgcttcgggatttatca	this study
ykfC$_{Cpn}$-C173A_rev	tgataaatcccgaagcatcaacacccggcttttccagact	this study
nlpC/P60$_{Ela}$-C193A_fow	gcacaagaccggaagcatccactcccctgaaagg	this study
nlpC/P60$_{Ela}$-C193A_rev	cctttcaggggagtggatgcttccggtcttgtgc	this study
nlpC/P60$_{Sne}$-C153A_fow	ggacctgtcaaggagtggatgcctcgggacttctt	this study
nlpC/P60$_{Sne}$-C153A_rev	aagaagtcccgaggcatccactccttgacaggtcc	this study
nlpC(ct127)/qPCR F	AACCATCGACACTATGCCTATTC	this study
nlpC(ct127)/qPCR R	TGAGAGCAGACAACAGCATTAG	this study
nlpC(ct127)/(pBOMBL)/F	aaagatcttcacacaggacatctgcATGCCGCACCAAGTCTTATTATC	this study
nlpC(ct127)_6xH/(pBOMBL)/R	acatatttgaatggtcgaccggtacttaatggtgatggtgatggtgAAAGAAGGCTTTTCTATTTTTAGGAAC	this study
nlpC(ct127) crRNA gBlock	tgtgaaagtgggtcttaagacgtcggtactgcatgtgacgcacgtagatcatgcaTTCACCGGTGGAGACGGTTTTCTTATAATGACACCTAATTTCTACTCTTGTAGATGTCCTCTTTGTGATGTCTCTACAAATAAAACGAAAGGCTCAGTCGAAAGACTGGGCCTTTCGTTTTATcaacagcggtctactgaatctgagctagtgcgtgatataattaaaattatattca	this study
non-targeting crRNA gBlock	tgtgaaagtgggtcttaagacgtcggtactgcatgtgacgcacgtagatcatgcaTTCACCGGTGGAGACGGTTTTCTTATAATGACACCTAATTTCTACTCTTGTAGATACCGAGTTGCCCGTTAAAGTACAAATAAAACGAAAGGCTCAGTCGAAAGACTGGGCCTTTCGTTTTATcaacagcggtctactgaatctgagctagtgcgtgatataattaaaattatattca	this study
^{sne}NlpC_F	GTTCCAGAACTTTTACGTTA	this study
^{sne}NlpC_R	AAAGAGCAAATCTTGTGTAT	this study
^{ela}NlpC_F	AGTTACAAAGCAATAGGAGT	this study

[ela]NlpC_R	CATCATCACATGATCAATC	this study
[sne]MreB_F	GTAAAACACCTCGTAAGATT	this study
[sne]MreB_R	GTACAGCAATTAAAATTTTG	this study
[ela]MreB_F	TTAAGGCTCTGATCAAAC	this study
[ela]MreB_R	CGATCAAAATAACCTCTT	this study

[a] in 5‘-3‘ direction.

Supplementary References

1. Xu Q, Abdubek P, Astakhova T, Axelrod HL, Bakolitsa C, Cai X, et al. Structure of the γ-D-glutamyl-L-diamino acid endopeptidase YkfC from Bacillus cereus in complex with L-Ala-γ-d-Glu: Insights into substrate recognition by NlpC/P60 cysteine peptidases. Acta Crystallogr Sect F Struct Biol Cryst Commun. 2010;66: 1354–1364. doi:10.1107/S1744309110021214
2. Pinheiro J, Biboy J, Vollmer W, Hirt RP, Keown JR, Artuyants A, et al. The Protozoan Trichomonas vaginalis Targets Bacteria with Laterally Acquired NlpC/P60 Peptidoglycan Hydrolases. MBio. 2018;9. doi:10.1128/mBio.01784-18
3. Ouellette SP, Blay EA, Hatch ND, Fisher-Marvin LA. CRISPR Interference To Inducibly Repress Gene Expression in Chlamydia trachomatis. Infect Immun. 2021 Jun 16;89(7):e0010821. doi: 10.1128/IAI.00108-21. Epub 2021 Jun 16. PMID: 33875479; PMCID: PMC8373233.
4. Brockett MR, Lee J, Cox J V., Liechti GW, Ouellette SP. A dynamic, ring-forming bactofilin critical for maintaining cell size in the obligate intracellular bacterium Chlamydia trachomatis. Infect Immun. 2021;89. doi:10.1128/IAI.00203-211
5. Baba T, Ara T, Hasegawa M, Takai Y, Okumura Y, Baba M, et al. Construction of E-scherichia coli K-12 in-frame, single-gene knockout mutants: The Keio collection. Mol Syst Biol. 2006;2. doi:10.1038/msb4100050
